# Rhizobacteria Exopolysaccharide: A Boon in Reclaiming Soil Fertility, Augmenting Plant Growth and Plant Stress Resilience

**DOI:** 10.1111/1758-2229.70296

**Published:** 2026-03-11

**Authors:** Aishmita Gantait, Sam A. Masih, Ann Maxton, Adriano Sofo, Rosangela Addesso

**Affiliations:** ^1^ Department of Genetics and Plant Breeding Sam Higginbottom University of Agriculture, Technology and Sciences Prayagraj India; ^2^ Department of Molecular and Cellular Engineering Sam Higginbottom University of Agriculture, Technology and Sciences Prayagraj India; ^3^ Department of Agricultural, Forestry, Food and Environmental Sciences (DAFE) University of Basilicata Potenza Italy

**Keywords:** agriculture, bacteria, biopolymers, microbial exopolysaccharides, stress resistance

## Abstract

Microbial exopolysaccharides (EPSs) serve multiple industrial and environmental purposes operating as complex biopolymers produced by bacteria and fungus, as well as by yeast and microalgae. The structural diversity of microbial EPS enables the biofilm formation, the stress resistance and the nutrient storage, comprising homopolysaccharides and heteropolysaccharides. Soil structure receives substantial improvement through EPS because the polymers help aggregate particles, retaining more water and trapping heavy metals, that results in enhanced soil fertility useful in sustainable agricultural practices. Moreover, through the presence of EPS‐producing bacteria, plants can establish beneficial connections with microorganisms that improve their tolerance to environmental factors, including salt exposure, drought conditions and extreme temperature changes. Such polymers find applications in the bioremediation and pharmaceutical fields because they present significant pharmacological properties, such as antibacterial, anti‐inflammatory activities and antioxidant behaviour. Their biodegradable nature and eco‐friendly properties make it eligible as a sustainable choice to replace synthetic polymers. This paper broaches the multiple ways how EPS improves plant wellness and enhances soil quality. Potential solutions emerge from microbial EPS research to address global challenges in agricultural sectors, biotechnological fields, and environmental management domains.

## Graphical abstract

Microbial exopolysaccharides (EPS) enhance plant stress tolerance and soil health by improving water retention, nutrient cycling, and resilience to salinity and heavy metals, offering a biodegradable and eco‐friendly alternative to synthetic polymers for sustainable agriculture.

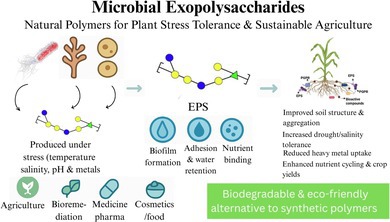

## 1 Introduction

Microorganisms, including bacteria, fungus, yeast and microalgae, generate interesting biopolymer exopolysaccharides (EPSs) (Kim et al. 2023). Microbial EPSs equip bacteria with a diverse set of compounds for ecological functions, including plant‐microbial associations and soil cohesion as well as plant resistance against stress (Wang et al. 2018a). In fact, the complex sugar chains structure of these polymers can form protective biofilms by binding with proteins and other elements, like lipids and metal ions, as well as extracellular DNA and organic and inorganic compounds (Addesso, Baldantoni, et al. 2023; Nadzir et al. 2021). The special characteristics of natural polymers, including chitin and chitosan—that exist in arthropod exoskeletons and specific fungal and yeast cell walls—enable them to become prominent elements in natural polysaccharide research due to their biocompatibility and biodegradability and fibre‐formation abilities (Rinaudo 2006). Chitin and chitosan mostly originate from ocean ecosystems and find application in the biomedicine, food system, pharmaceutical and cosmetic industries, whereas EPSs derive from microorganisms that manufacture these substances because of environmental stressors, such as temperature fluctuations, salts and pH changes alongside chemical substances and radiative conditions (Kumari et al. 2023). Therefore, EPSs function depends mostly on the structural components they contain, being influenced by the natural habitat of their host microorganisms. They serve a dual purpose in cellular adhesion and protective mechanisms by aiding survival and adaptation in unfavourable growth environments, though their shorter production duration than other life forms (Salimi and Farrokh 2023). In fact, microorganisms generate EPSs for environmental adaptation, enabling them to survive across low temperatures, high salt conditions, and temperature variations from freezing to thawing (Osemwegie et al. 2020). Moreover, they work as soluble or insoluble molecules that exhibit multiple properties unlike typical plant polysaccharides (Nadzir et al. 2021), raising growing interest due to their potential use in bioremediation systems, drug delivery methods and plant resistance enhancement against environmental aggressors (Netrusov et al. 2023).

Exopolysaccharides (EPS) are important in improving plant health and sustainable agriculture through improving the soil structure and nutrient cycling and resilience to stress. Microorganisms produce these high molecular weight polymers, which play a vital role in trauma generation and stability of soil biofilms that are fundamentals to support soil health and functionality (Zhang, Wang, et al. 2024; Zhang, Wu, et al. 2024). Rhizobacteria exopolysaccharides stimulate plant growth through triggering biofilm formation, host colonisation, and stress resistance in the environment. They are essential in sustainable agriculture because they enhance crop productivity and soil fertility via processes such as phytoremediation and stress alleviation (Bramhachari et al. 2018). EPS make nutrients easy to trap and water more resistant to environmental factors like water scarcity and salinity, which promotes the growth of plants and their tolerance to environmental degradation (Bramhachari et al. 2018; Zhang, Wang, et al. 2024; Zhang, Wu, et al. 2024). Moreover, EPS are also used in the process of detoxifying the contamination of pollutants and pathogen nipping of soil, which also stimulates crop yields further (Awasthi et al. 2017). With the growth of populations and the demand of agriculture practices, exploitation of EPS with microbial interactions is one of the promising approaches to sustainable agricultural practices, where the health of the environment and food security are guaranteed (Awasthi et al. 2017).

EPSs display many advantages as compared to synthetic polymers together with their remarkable ability to thrive under demanding environments. Seventy percent of EPSs and other natural polymers recycle in the environment within months due to their natural origin, so they are both biodegradable and environmentally friendly (Angelin and Kavitha 2020). For this reason, EPSs earn the status as sustainable alternatives for different scientific and industrial operations. Moreover, EPSs helps in reducing the effect of stress experienced by plants, from drought to salinity and harsh temperatures, affecting their development and agricultural yield (Pacholak et al. 2021). EPSs show promising potential in transformative scientific development because they push advances in both industrial fields and create sustainable useful solutions.

## 2 EPSs

The term exopolysaccharide was coined by Sutherland in 1972 and used for high‐molecular‐weight carbohydrate biopolymers produced by microorganisms (Osemwegie et al. 2020). Microbial EPSs have become the natural polymers that are vital to industrial applications, health and environmental management (Bhagat et al. 2021). They are created to exchange energy in response to signals from the environment (Flemming and Wingender 2001). Comprising many biomolecule kinds, they include polysaccharides, sugars, structural proteins, enzymes, amino, sugars, nucleic acids, lipids, pyruvates, glycoproteins, lipids, extracellular DNA and some humic compounds (Mishra and Jha 2013). Thanks to the differences in their architecture and characteristics, they can work well in several fields. Some of the EPS‐producing bacteria belong to different Gram‐negative phylogenetic classes, including *Alphaproteobacteria*, that include *Acetobacter, Gluconobacter*, *Gluconacetobacter*, *Komagataeibacter*, *Kozakia*, *Neoasaia*, *Agrobacterium*, *Rhizobium* and *Zymomonas* genera; *Betaproteobacteria* class that include Gram‐positive bacteria; *Bacilli* class, that includes *Bacillus*, *Paenibacillus*, *Lactobacillus*, *Leuconostoc* and *Streptococcus* genera, *Clostridia* class, including *Sarcina* sp., *Actinomycetes* including *Bifidobacterium*, *Rhodococcus* genera and others (Díaz‐Cornejo et al. 2023; Wünsche and Schmid 2023; Netrusov et al. 2023). The most frequently reported EPSs are xanthan, that belongs to *Xanthomonas*, dextran from *Leuconostoc*, *Streptococcus*, *Lactobacillus*, alginate by *Azotobacte*r and *Pseudomonas*, curdlan by *Alcaligenes faecalis* and *Rhizobium*, from class *Alphaproteobacteria* there are *Radiobacter* and *Agrobacterium* sp., from *Sphingomonas* and *Pseudomonas* the gellan, from *Streptococcus* sp., the hyaluronan, from *Bacillus* sp., the levan, *Paenibacillus* sp. *Halomonas* sp., *Zymomonas* sp., bacterial cellulose from *Komagataeibacter* sp. and so on (Netrusov et al. 2023). There are also known polysaccharides, for instance, fucogel from *Klebsiella pneumoniae* , clavan from *Clavibacter michiganensis* , fucoPol from *Enterobacter* sp., and kefiran from *Lactobacillus kefiranofaciens* (Barcelos et al. 2019; Xiao et al. 2022). EPSs produced by bacteria are superior to those extracted from plants, animals and algae, including cellulose, starch, pectin, glycogen, chitin, alginates, fucoidan, carrageenan and agar (Netrusov et al. 2023; Grauso et al. 2023). The significance of bacterial EPSs in a diversified range of biological and industrial applications is mainly attributed to structural variance and functional capability of these polymers. They are essential in the development of biofilm, improving the tolerance of microbial community to stress and act as repositories for nutrients (Jyoti et al. 2024). EPSs from both eukaryotic and prokaryotic microorganisms are multitalented, find application in various industries working in food, pharmaceuticals, and environmental remediation sectors, primarily due to their biodegradable property and distinct physicochemical characteristics (Mouro et al. 2024). Studies have shown that EPSs possess interesting pharmacological properties such as antioxidants, antimicrobial and anti‐inflammatory properties, that could be important in the therapeutic application, for instance, in inflammatory bowel disease. Additionally, the fermentation process in EPS production by different techniques and environmental factors can improve yield and functional properties that make them useful in sustainable biological processes in biotechnology.

The EPSs produced by bacteria serve a vital function in plant–microbe relationships, that specifically affect plant growth‐promoting rhizobacteria, that live close to or on the root of the plants. EPSs assist these bacteria to adhere to the roots, form protective biofilms and survive stressful situations such as drought or diseases thereby enhancing the health of the plants, uptake of nutrients and environmental stress resistance indirectly (Morcillo and Manzanera 2021). Various *Pseudomonas* and *Azospirillum*, as well as *Azotobacter* and *Bacillus* bacteria species, among others promote plant development while taking nutrients and producing phytohormones and suppressing diseases (Fan and Smith 2021). They work as plant‐signalling chemicals to activate defence mechanisms in plants as well as activate systemic pathogen resistance responses. Moreover, they help to enhance soil structure while increasing water retention capacity, thus making nutrients and water more accessible to plants during their growth cycle (Addesso, Sofo, and Amato 2023; Massa et al. 2022).

Many microbial EPSs are well known to be involved in plant‐microbe interactions contributing to overall plant health and productivity in agriculture. Synthesised by several microorganisms, they have a positive impact on soil properties because of the nutrients' storage ability, improving microbial communities that are crucial for plants to grow, especially under conditions of abiotic stress (Akhtar et al. 2024). For example, research has confirmed that EPSs enhance crop productivity, with soybean and maize yields growing by 57.7% and 44.8% when used in combination with nitrogen‐fixing bacteria (Chaudhary et al. 2024). Further, it is reported that EPSs have multiple pharmacological activities such as antioxidants and antimicrobial properties that enhance plant resistance and vigour. Microbial taxa like *Paraburkholderia phytofirmans* (PsJN) show that EPSs synthesis is essential for efficient plants and stress adaptation as it relies on EPSs for effective colonisation of plant tissues, especially under drought conditions (Fu and Yan 2023). Thus, EPSs are the direction for the development of sustainable agriculture and improving the stability of crops in unfavourable conditions.

EPSs exist in two separate forms after synthesis, capsular and slime, performing distinct protective functions for the cells. Capsular polysaccharides form a protective cell surface barrier (Rawat et al. 2024). Free slime polysaccharides exhibit either weak surface binding or exist as viscous or gelatinous substances to perform multiple functions including adhesion, colonisation and ecological stress defence (Angelin and Kavitha 2020).

Exopolysaccharides are multifaceted biomolecules that are made up of carbohydrate polymers, which may be diverse in terms of their structural composition and chemical constituents. The biochemical composition of EPSs differs depending on their microbial origin (bacterial, fungal or algae). The biochemical composition of bacterial EPSs includes either homopolysaccharides or heteropolysaccharides (Mouro et al. 2024). EPSs homopolymers (Table 1), unbranched or branched, contain a single monosaccharide type linked by glycosidic bonds to form repeated units primarily composed of glucose together with fructose units (Díaz‐Cornejo et al. 2023). The EPS family of α‐D‐glucans includes dextran and alternan together with reuteran;β‐D‐glucans group contains bacterial cellulose and curdlan, while fructans include levan and inulin (Waoo et al. 2023). Complex structures, along with levan and inulin, form part of the heteropolymer family of EPSs (Gupta et al. 2019). The composition of heteropolysaccharides takes on a complicated arrangement that unites various monosaccharides such as glucose, fructose, galactose, mannose, rhamnose, fucose, arabinose, xylose, N‐acetylglucosamine and uronic acids and multiple non‐carbohydrate groups (Table 2) (Netrusov et al. 2023; Kaur and Dey 2023; Xiao et al. 2022; Nadzir et al. 2021). Various heteropolysaccharide molecules comprise xanthan gum along with alginate, hyaluronic acid, kefiran, gellan and additional types (Silva et al. 2009). The fungal EPSs are usually rich in glucans and mannan and other polysaccharides that have simpler or more repetitive structures (Mahapatra and Banerjee 2013). These polymers primarily promote fungal growth and protection and are immunomodulatory and antioxidant which can find applications in pharmaceutical and nutraceutical applications. On the other hand, algal EPSs are commonly sulphated polysaccharides such as carrageenan, agar, alginates and fucoidan (Kraan 2012). They are designed to gel, hydrate and protect under water conditions. The widely used application of algal EPSs in food, cosmetic and pharmaceutical industries is as a thickener, stabiliser and emulsifier.

**TABLE 1:** Types of homopolysaccharides.

EPS	Producer organism	Function	Application	References
Dextran	It is composed of glucose and produced by Lactic acid bacteria, that belong to *Leuconostoc*, *Streptococcus*, *Weisella*, *Lactobacillus* and *Pediococcus* genera produce the compound Dextran.	It exhibits multiple functionalities, including thickening ability, viscosity regulation, stabilising effect and emulsifying action. It provides anticancer properties and antibacterial effects and antifungal abilities despite being biodegradable and biocompatible and non‐toxic. It shows high aqueous solubility.	In bakery, it improves moisture retention, softness and texture of the products. In ice cream and jams‐ stabilises, it thickens and prevents crystallisation of products. Used in pharmaceutical and cosmetics industries. Used as chromatographic medium.	(Nadzir et al. 2021; Tabernero and Cardea 2020; Waoo et al. 2023; Yildiz and Karatas 2018)
Alternan	It is a α‐D‐glucans produced by *Streptococcus salivarius* , *Leuconostoc citreum* and *Leuconostoc mesenteroides* .	It is soluble in water. It has a low viscosity. It displays exceptional resistance against microorganisms and enzyme action from mammalian organisms.	It functions as a prebiotic substance while providing texture control and serving as a lower calorie alternative to fat or oil for meal ingredients, thus making it a useful food ingredient in the commercial food sector. Used as lipid‐substitute texturizers for cosmetic preparations like ointments and lotions. When added to the wet end of the papermaking process, it increases the dry strength of the finished product.	(Chen et al. 2021; Basiri 2021; Gangoiti et al. 2020; Zikmanis et al. 2020)
Reuteran	The enzyme reuteransucrase from *Limosilactobacillus reuteri* generates this water‐soluble α‐glucan.	Non‐Newtonian behaviour in aqueous solution and water solubility. Milky‐white opalescence is present.	When applied in baking, it results in improvements in both texture and quality of bread products. The addition of this compound results in improved quality for gluten‐free and sorghum sourdough bread by providing longer durability and tenderness with prebiotic benefits.	(Basiri 2021; Chen et al. 2021; Tabibloghmany and Ehsandoost 2014)
Bacterial cellulose	Produced by *Gluconacetobacter*, *Rhizobium*, *Sarcina*, *Agrobacterium* and *Salmonella*.	Its unique properties include nonallergic and nontoxic benefits. It possesses special qualities like large surface area, high purity, high yield, crystalline, high polymerisation, tensile strength, lightweight nature, water‐holding capacity, transparency, flexibility, biodegradability, biocompatibility and renewability.	Bacterial cellulose works as a flexible natural polymer that can create different products from food to medicine and manufacturing. Biomedicine uses this as part of tissue engineering tools, wound treatment supplies, and medicine dosage control systems. Bacterial cellulose serves as a material in cosmeceutical face masks helps deliver bioactive materials and keeps the skin properly hydrated. Bacterial cellulose functions as a stabiliser for emulsion mixtures in technology.	(Bianchet et al. 2020; Choi et al. 2022; Lahiri et al. 2021)
Curdlan	The microorganisms *Agrobacterium*, *Rhizobium*, *Bacillus* and *Cellulomonas* generate this neutral and acidic linear glucan as a secondary product under conditions with limited nitrogen supply.	Dissolves in salt‐water mixture it remains insoluble with plain water while showing soluble behaviour with alkaline solutions. It is indigestible and does not have any taste, colour or odour. It reduces inflammation while raising cytokine activity and works against cancer development.	Medical research shows specific advantages for controlling immune reactions, packaging drugs, and strengthening composite dressing materials. It helps bone develop and joins mesenchymal cells together. Curdlan and its modifiers have been effectively applied to create drug delivery technologies that hold medications in place. Food manufacturers often use it to make their manufactured foods thicker and more stable. They also apply it to packed meats, processed noodles, sauces, frozen dishes, and yogurt to enhance their textures.	(Anane et al. 2017; Chaudhari et al. 2021)
Levan	Through levansucrases multiple bacteria, such as *Acetobacter, Bacillus*, *Brenneria*, *Geobacillus*, *Halomonas*, *Lactobacillus*, *Zymomonas* and *Saccharomyces* synthesise this fructose‐based homopolysaccharide.	It acts as a water and oil‐based polymer that stays undissolved in standard organic solvents. It produces strong films at high temperatures while staying resistant to water uptake at normal room temperature due to its low viscosity properties. It is an effective compound that delivers various health advantages such as protecting against cancer, slowing down antioxidants, preventing bacteria growth, reducing inflammation and cholesterol levels, shielding from radiation damage, improving immunity and offering digestive system support.	Levan proves very useful in both food and biomedicine industries plus cosmetics and pharmaceuticals. Its diverse properties show great potential in medicine through blood plasma replacement systems combined with weight reduction drug development and anticancer treatment of elevated blood sugar levels. It shows treatment benefits against human health diseases, like cancer, diabetes, heart problems, and it has a special ability to fight neuroblastoma and osteosarcoma cell growth because of its recognition of GLUT5 receptors.	(Jadaun et al. 2019; Basiri 2021)
Inulin	It is a fructo‐oligosaccharides and are synthesised by *Streptococcusmutans*, *Limosilactobacillus reuteri*, *Leuconostoc citreum* and *Lactobacillus johnsonii* .	It enhances water viscosity. It offers multiple health advantages that help the body absorb calcium and fight harmful bacteria while showing natural defence against harmful molecules.	It replaces fats and carbohydrates and lowers the calorie content of meals. It serves a prebiotic purpose. It functions as a cryoprotectant and stabilising agent for foods. It is useful for medical treatment as it serves as a transport method for medical treatments in colon problems and helps prevent cancer, mainly colon cancer. It improves bowel movements while reducing the number of people who develop irritable bowel diseases.	(Gupta et al. 2019; Teferra 2021)
Pulluan	It is a soluble polymer that arises through fermentation by *Aureobasidium pullulans* that appears naturally in soil. Other microorganisms that can produce pullulan include *Cytaria* spp., *Teloschistes flavicans*, *Rhodotorula bacarum* and *Cryphonectria parasitica* .	It is a white powdery substance that shows no toxicity and breaks down naturally while matching body tissue and having no odour, taste or oxygen permeability. Water dissolves pullulan well, and it also absorbs a large amount of water. It shows high heat resistance alongside strong binding capabilities and sturdy mechanical performance under various pH levels. It has natural properties, including strong antioxidant protection and safe bioactivity that do not harm human tissues.	It is employed in tissue engineering, biomedical imaging, plasma expanders, nasal vaccination adjuvants, vaccine formulations, skincare products, and cosmetic formulations besides serving as a carrier for gene and medication delivery. It is utilised as a dietary fibre in low‐calorie meals, as well as in food coating, edible films, packaging films, prebiotics, thickening agents and carriers of flavours and antibacterial chemicals. It is employed in the fields of electronics, energy and waste remediation and analytical methods.	(Prajapati et al. 2013; Cruz‐Santos et al. 2023; Wani et al. 2021; De Souza et al. 2023)
Mutan	Oral bacteria species, especially *Streptococcus mutans* and *Streptococcus sobrinus* produce Mutan.	It is sticky, colourless and water insoluble.	It stops plaque growth and prevents tooth decay. It serves to absorb heavy metal contamination besides its dental healthcare applications.	(Boddapati et al. 2020; Yu et al. 2018)

**TABLE 2:** Types of heteropolysaccharides.

EPS	Producer organism	Function	Application	References
Xanthan gum	It is a multipurpose heteropolysaccharide is produce by *Xanthomonas campestris* .	High viscosity, water solubility, non‐toxicity, resistance to environmental influences, biodegradability, cost‐effectiveness, antioxidant benefits, antibacterial and antitumoral qualities, and viscosifying and stabilising qualities.	It functions both as a framework and drug carrier system. Healthcare teams use it as an immune‐strengthening agent while creating body parts and wounds. Staff rely on it for needle‐free medicine delivery within joints and to heal skin damage. Manufacturing companies use it to make personal care products along with soap products and insect protection items plus eco‐friendly cleaning items. It helps stabilise food emulsions while thickening the texture and preventing crystal growth in different food products. It plays critical roles in different printing approaches and helps retrieve oil while adjusting drilling liquid movement.	(Silva et al. 2009)
Alginate	It is a linear heteropolysaccharide, produced by bacteria like *Pseudomonas aeruginosa* and *Azotobacter vinelandii* .	Water‐soluble; biocompatible and biodegradable; hydrogel‐forming; water‐holding; viscosity‐regulating; and stabilising qualities.	Cells, growth factors, and medications can all be encapsulated by it. Biomedical practitioners use this when creating engineered tissue blocks. For bone engineering, it works as a support device to hold osteoinductive factors. Skin products use this substance as both an ingredient and a thickening/pudding agent. It offers three essential functions as a support component and thickening solution, along with serving as a protective package for materials.	(Tabernero and Cardea 2020; Waoo et al. 2023; Nadzir et al. 2021)
Hyaluronic acid	Several bacterial types like *Streptococcus equi* , *Streptococcus equisimilis*, *Streptococcus pyogenes* and *Streptococcus thermophilus* produce this acidic heteropolysaccharide.	High water retention capacity; non‐immunogenic, biocompatible, and biodegradable; moisture‐absorption qualities; viscoelastic; improve cell adhesion and proliferation; create gel‐like, non‐Newtonian solutions; have anti‐inflammatory qualities; adaptable to biological systems; regulation of angiogenesis.	In pharmaceuticals, it serves as a hydrating ingredient. It is used to create artificial tear medicines and solutions. Scientists use this material to fight cancer. The substance helps cells move toward wounds while promoting natural blood vessel growth and recovery. Research teams use it as a base for creating fabricated tissues. It works to treat patients who have osteoarthritis. Medical teams apply it as a joint lubricant to copy synovial fluid motion, while doctors employ it to act as artificial eye fluid during eye treatments. Hyaluronic acid helps stop bowel connections from developing in abdominal surgeries. The medical field uses it as protective outer layers over surfaces. It is an ingredient in food.	(Serra et al. 2023; Don and Shoparwe 2010)
Kefiran	It is produced by *Lactobacillus delbrueckii subsp. bulgaricus* together with *Lactobacillus kefiri* , *Lactobacillus parakefir*, *Lactobacillus kefiranofaciens* and *Lactobacillus kefirgranum* .	Water soluble, semi‐crystalline, hydrolysis‐resistant, capable of forming gels and films, water vapour barrier, antibacterial, antioxidant, anticancer and mucosal adjuvant qualities, as well as biodegradable and biocompatible.	Tissue engineering and wound dressings with regulated medication and probiotic administration represent two major applications of this substance. The utilisation of this material extends to edible bio‐films together with biodegradable food packaging. It is applied in fermented dairy products as a thickener while maintaining food stability.	(Tan et al. 2020; Gentry et al. 2023)
Gellan	Produced by *Sphingomonas paucimobilis* .	Low mechanical resistance, high polyelectrolyte content, resistance to enzymatic degradation, thermos‐reversible gel formation, decreased sensitivity to pH fluctuations and insoluble in cold water.	Food manufacturers use this ingredient to regulate texture and flavour release in sweets, jams, and ice creams besides using it as a stabiliser along with being a binder and thickener and gelling agent. Vitamin C and heat‐delicate molecules find an encapsulating solution in thickeners that double as transportation vehicles. This substance mainly finds two main applications in pharmaceutical products by aiding dissolvable pill tablets and managing medication delivery mechanisms. It assists cells in differentiating and propagating while facilitating bonding interactions between them. It serves as a water flocculent while being used for paper coatings. It is a natural tool to purify polluted groundwater reservoirs alongside defiled earth materials.	(Viswanathan et al. 2023; Mouro et al. 2024)
Emulsan	Produced by *Acinetobacter venetianus* and *Acinetobacter calcoaceticus* .	It has qualities such as amphiphilia, chemical and biological adaptability and bio‐emulsifying capabilities.	For industrial and environmental applications, it is used as stable hydrocarbon‐in‐water emulsions. It is employed in the delivery of drugs. The food industry uses bio‐emulsifier properties of this product during production processes. Toothpaste and shampoo together with lotion and washing creams use this ingredient as one of their components. It serves as an important vaccine stimulant.	(Ansari et al. 2023; Castro et al. 2008; Yi et al. 2019; Mouro et al. 2024)

Bacterial EPSs are biosynthesized by a set of enzymatic reactions, which degrade simple sugar precursors into more complex polysaccharides. It normally starts with the absorption of sugars such as glucose or fructose, which undergo the phosphorylation stage into sugar nucleotides (e.g., UDP‐glucose). Such active forms are subsequently polymerised by glycosyltransferases to create replicating sugar units which are linked together to create high‐molecular‐weight chains (Czaczyk and Myszka 2007). Synthesis of EPS can also be through various pathways depending on the bacterial species, and this may be the Wzy‐dependent pathway, the ABC transporter‐dependent pathway, or the synthase‐dependent pathway (Schmid 2018). These pathways control the structure, branching, and functional characteristics of EPSs that enable bacteria to respond to environmental cues and stressors by changing the polymer composition.

## 3 EPSs in Improving Soil Quality and Plant Growth

**FIGURE 1:**
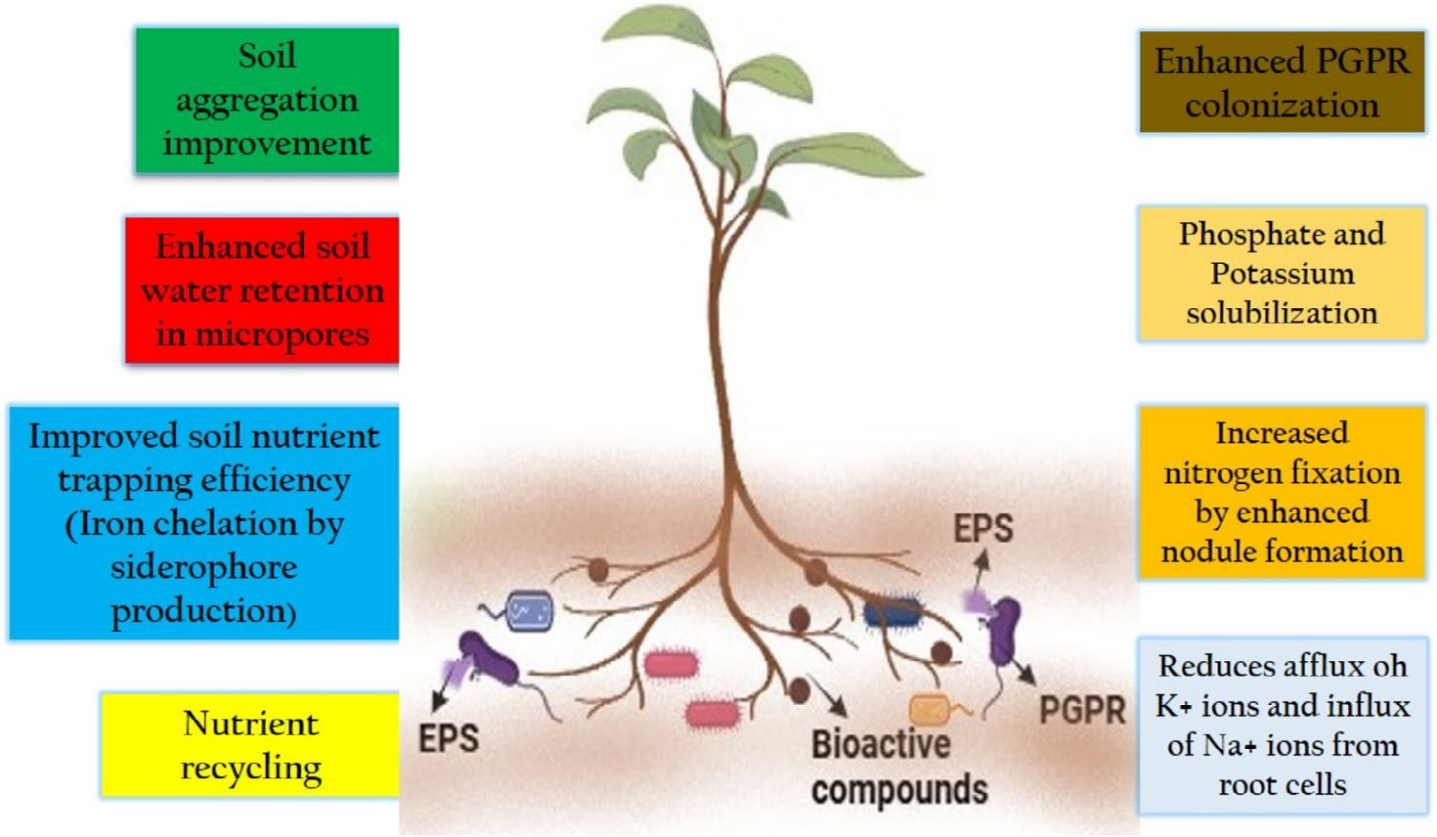
Rhizobacterial exopolysaccharides for soil health improvement and plant growth augmentation.

Soil science focuses intensively on EPSs that are complex carbohydrates produced by microorganisms because these substances powerfully improve soil quality (Figure 1). Soil health and fertility increase through the secretion of extracellular polymeric substances by bacteria and fungi, that generate multiple advantages to modify both physical, chemical and biological soil properties (Walshire et al. 2024). The main role of EPSs in soil involves enhancing the soil aggregation and structure. EPSs enhance soil structure in combination with enhanced fertility by three key aspects that include improving aggregation of soil components and nutrient access, as well as altering microbial community actions (Figure 1) (Chaudhary et al. 2024). The polysaccharides serve as a binding agent to connect soil particles, that produce a porous stable soil structure. The better soil structure created by EPSs leads to enhanced water infiltration and soil aeration and decreases erosion risk, that is essential for agricultural system sustainability (Regar 2023). EPSs act as a key component to develop and preserve soil aggregates, that serve as basic elements for sustaining fertile soil to establish an environment suitable for microscopic organisms that create diverse microbial communities. Additionally, they improve soil structure by establishing a cyclic positive loop, hence showing great potential in enhancing soil water‐holding properties as one of their main contributions to soil quality (Carezzano et al. 2023). The EPSs produced by bacterial strains *Bacillus* sp. and *Rhizobium* contribute to microaggregates formation in soil enhancing its stability along with improving water retention capacity (Walshire et al. 2024). EPSs act as important heavy metal immobilisers that reduce their availability and toxicity to plants based on the observed lead and cadmium uptake decrease in lettuce and pakchoi crops (Liu et al. 2024). Through their ability to form stable metal complexes, *Bacillus* strains Z23 and Z39 reduced cadmium and lead uptake in lettuce to 88.6% and 93.2%, respectively (Zhang, Wang, et al. 2024; Zhang, Wu, et al. 2024). The EPSs of microbial origin show a different repertoire of properties for boosting soil quality, especially during heavy metal cleaning operations and soil structure reinforcement efforts. Soil contaminated with lead received exceptional treatment from the marine bacterium *Micrococcus antarcticus* HZ because it removed from 43.3% to 63.5% of lead, while boosting the growth of pakchoi plants (Liu et al. 2024). Soil health enhances through EPSs application because the method boosts both soil enzyme functioning and nitrogen availability, enabling increased populations of beneficial microorganisms responsible for nutrient cycling (Chaudhary et al. 2024). The multiple actions provided by EPSs establish their potential as a sustainable strategy for improving soil health in crop production rates, particularly in polluted environments and degraded lands (Niranjani et al. 2023). The inactive metabolites produced by different microorganisms strengthen both soil aggregation and water retention capabilities that sustain agriculture notably in dry conditions (Niranjani et al. 2023). The distinctive traits of EPS synthesis from microbial strains have a fundamental impact on enhancing soil health alongside plant growth effectiveness across different environmental demands.

## 4 Role of EPSs in Plant Tolerance to Environmental Stresses

**FIGURE 2:**
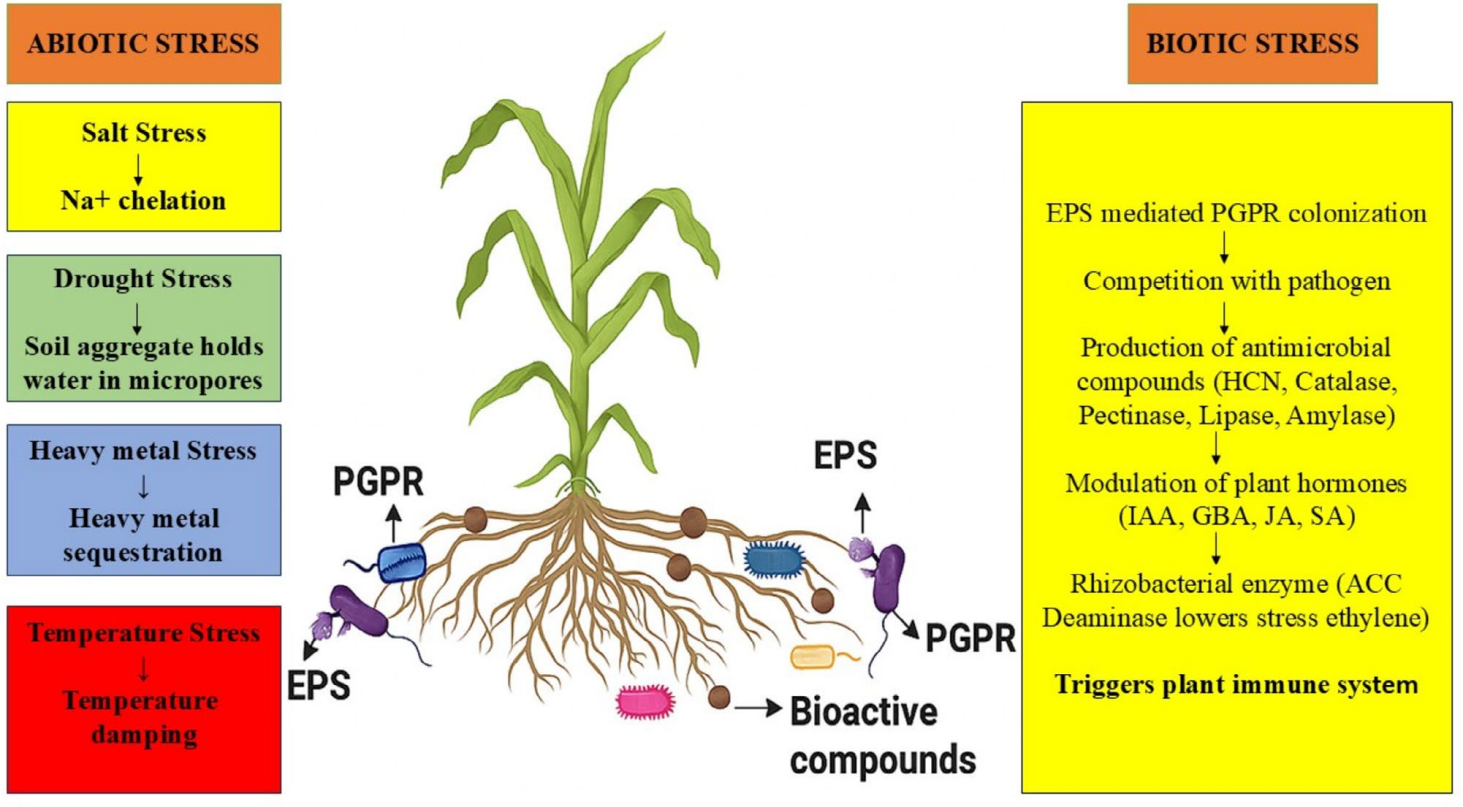
Rhizobacterial EPSs mediated abiotic/biotic stress tolerance.

Plants develop increased tolerance against environmental stress through the application of microbial EPSs (Figure 2). EPSs synthesis occurs primarily during the late exponential or stationary phase of microbial growth in response to multiple environmental stress conditions (Figure 2) made up of extreme temperature, salinity, unfavourable pH levels, osmotic stress, exposure to radiation, chemical agents, antibiotics, heavy metals and oxidants (Table 3) (Mouro et al. 2024). The diverse microorganisms present in soil need EPSs to thrive in adverse conditions, particularly antibiotic exposure and variations in pH levels along with osmotic stress and immune system defence mechanisms of the host (Fan et al. 2019). The EPSs layers produced by these polymeric substances show high viscosity and behave as hygroscopic and aerophytic materials that hold more water than their environment. Through this property, these substances maintain effective control over the microenvironment while offering protection to microbial cells against diverse stressors (Rana and Upadhyay 2020).

**TABLE 3:** Role of bacterial EPS in plant tolerance to various stresses.

Sl. No.	Stress	Role of bacterial EPS in plant tolerance to various stresses	References
1	Drought stress	Bacterial EPSs assists plants to withstand droughts through the enhancement of water retention in the soil. They can hold moisture because they are hygroscopic, and this minimises the loss of water around the roots. EPSs also enhance soil aggregation, therefore, allowing plants to be able to access water easily. They also help to establish biofilms on root surfaces which maintain the attachment of beneficial microbes and aid in the growth of plants in dry soils.	(Fan et al. 2019; Prihatna and Yan 2024)
2	Salinity stress	EPSs assist plants to control salt stress by binding the sodium ions and other salts, and thereby they do not accumulate in the tissues of the plants. This lessens the toxicity of salinity. They also stabilise cell membranes of plants stopping the leakage of electrolytes. Additionally, nutrient uptake is enhanced through EPSs which adjusts the root environment that enables plants to absorb vital nutrients even when subjected to saline conditions.	(Labella et al. 2024; Rana and Upadhyay 2020)
3	Temperature stress	EPSs also safeguard plants in cold conditions because they keep the plants hydrated and do not allow the formation of ice crystals in the interior cells, which may damage the plant. During heat stress, EPSs stabilise the cell membranes and proteins which enable the plants to endure high temperatures and also oxidative stress. Biofilms which EPSs create are used to resist temperature changes and protect roots and microorganisms.	(Rana and Upadhyay 2020)
4	Heavy metal stress	EPSs contain cadmium and lead which are very toxic to plants by attaching itself to such toxic metals and making them less available. This will avoid the occurrence of harmful metals that may interfere with the metabolism of plants. The complexes of metal and EPS immobilise toxins, which protect the root tissues and enhance the development of plants in the contaminated soils.	(Bhagat et al. 2021)
5	Nutrient deficiency	EPSs make major nutrients such as phosphates and iron more accessible through attachment to them or change of soil pH. They also promote the development of useful microbes which help in cycling of the nutrients to make sure that plants access the necessary elements even in nutrient depleted soils.	(Naseem et al. 2018)
6	Biotic stress	EPS forms a protective biofilm over roots of the plant that makes a physical barrier between the entry of pathogens into plant tissues. They also initiate the immune response in plants, which is the activation of the defences by plants against infections. Also, EPSs give nourishment to beneficial microorganisms that outcompete the harmful pathogens thereby lowering chances of disease.	(Dimkpa et al. 2009)

The EPSs that plants acquire from their root‐associated microbes generate protective layers by encasing plant cells enabling them to manage unfavourable growth conditions. Scientific evidence demonstrates that EPSs successfully reduce salt stress effects on plants during their developmental period (Labella et al. 2024; Rana and Upadhyay 2020). Plants become more resilient to dry conditions through EPSs production because these substances boost water retention while decreasing moisture loss (Fan et al. 2019). EPSs work as vital agents to reduce the negative effects extreme temperatures have on plants. The plant can maintain survival throughout freezing environments and resist cold stress by employing exopolysaccharide molecules that regulate membrane lipids and establish balanced ion levels (Rana and Upadhyay 2020). Using EPSs enables plants to deal with nutrient deficiencies because they improve nutrient availability and promote better nutritional absorption. The application of EPSs enables plants to gain enhanced resistance toward biotic threats because these compounds shift the root microbiome and kill harmful disease‐producing organisms (Dimkpa et al. 2009). EPSs create supplementary plant stress tolerance through their ability to modify the composition and activity dynamics of the root microbiome. *Pseudoalteromonas* (SM20310) produces bacterial EPSs, that protect the host strain to survive at low temperatures and in high salt situations and during freeze‐thaws (Liu et al. 2013). The environmental stress tolerance of plants seems to benefit substantially through the EPS secretions released by soil microbes (Wang et al. 2018). *Paraburkholderia phytofirmans* (PsJN) depends on EPSs for its ability to boost pea plant resistance against drought stress through establishment of successful plant colonisation and improved seed germination and seedling development (Prihatna and Yan 2024).

The application of bacterial EPSs binds NaCl and creates benefits for plant tolerance to stress conditions through improved membrane stability and reduced electrolyte leakage plus enhanced chlorophyll content that results in boosted growth in *Triticum aestivum* after bacterial consortium inoculation (Thakur and Yadav 2024). They promote growth alongside chlorophyll content elevation and enhanced antioxidant enzyme function. EPSs reduce detrimental substances while controlling phytohormones to improve salt stress tolerance when applied to tomatoes under salt stress conditions (Chen et al. 2023). The protective action of bacterial EPSs toward microbial cells boosts plant development and adaptability. Few plant growth‐promoting rizhobacteria (PGPR) strains release antimicrobial agents that enhance plant systemic defence responses and produce phytohormones that modify plant structural development and defensive and hormonal processes. PGPR enhances plant fitness by helping the plant access fundamental resources when they dissolve mineral phosphates and other vital nutrients (Xiong et al. 2023). Bacterial bioemulsifiers and biosurfactants when used on soil lead to better water retention capability and reduced water repellence of soil in dry areas where organic matter and water remain scarce (Addesso, Sofo, and Amato 2023; Raddadi et al. 2018). Plant‐derived molecules in combination with exudates trigger structural changes in the root microbiome, so it attracts EPS‐producing microbial species that strengthen plant resistance to environmental stress factors (Pascale et al. 2020).

## 5 Limitations and Future Aspects

The agricultural prospects of exopolysaccharides (EPSs) for plant‐microbe interaction enhancement exist with many research boundaries and promising future investigation directions. Under the production of microorganisms, including *Paenibacillus polymyxa* , EPSs show essential plant‐promoting properties by boosting root mineral acquisition and generating a protective bacterial habitat around plant roots (Huang et al. 2024). EPSs implementation exists as limited because their structures are complicated, and their production outcomes fluctuate according to environmental situations. EPSs remain underutilised due to issues involving their high production costs, along with a technical complexity of industrial‐scale manufacturing and purification processes (Mouro et al. 2024). The complexity of the isolation and purification process is one of the major constraints. EPS is extracted in several steps such as cell removal, solvent or salt precipitation, dialysis, chromatography or enzyme treatment and final storage or use in drying process. These processes could be costly, laborious and time‐consuming, especially where highly pure products are needed in sensitive usage. Moreover, the EPS recovery methods must be modified depending on the microbial strain, level of purity required and intended use. It is not easily produced and implemented in industries on a large scale due to this variability. EPSs present extraordinary scope in sustainable agriculture since they represent environmentally friendly alternatives to synthetic polymers despite facing some production obstacles (Netrusov et al. 2023). The next stage of research should concentrate on process optimization and genetic and metabolic EPS biosynthesis pathway examination together with discovering new EPS‐producing microorganisms, especially those found in extreme environments that display rare protective biological features (Huang et al. 2024). Application of EPSs in sustainable agricultural practices can be improved through studies of their interactions with plant‐associated microbiomes for biocontrol objectives and stress reduction and nutrient delivery functions (Kaur and Saxena 2024). The advancement of EPSs in agricultural applications depends on the solution of existing obstacles and knowledge expansion in upcoming areas.

## 6 Conclusions

Exopolysaccharides (EPSs) produced by various microorganisms possess complex structural arrangements and multiple functions leading to their extensive industrial, medical and agricultural worth. The biochemical structure of EPSs, either as homopolysaccharides or heteropolysaccharides, enables them to perform biofilm formation with stress tolerance functions and nutrient storage capabilities. Limited concentrations of EPSs contribute positively to both the development of plant‐microbe bonds and the improvement of soil texture, and the enhancement of plant health and stress tolerance. EPSs demonstrate therapeutic utility in medical and pharmaceutical applications because they contain antioxidant, antimicrobial and anti‐inflammatory properties. Continued research into microbial EPSs expands opportunities to use this discovery in sustainable agriculture and environmental protection. The importance of microbial EPSs extends to both plant protection against stress and the enhancement of soil conditions, and the provision of eco‐friendly technological solutions for industries. The biopolymer compounds produced by bacteria, fungi and microalgae show exceptional structural complexity and performance abilities that work as protective components in environments with high salinity and dryness conditions, together with heavy metal contamination. Soil fertility under abiotic stress conditions improves, and plant development strengthens because of EPSs, which enhance soil aggregate stability, save water, and deliver nutrients effectively. EPS‐producing bacteria strengthen plant stress survival by simultaneously colonising roots while improving nutrient uptake, which leads to better crop growth together with stable ecological conditions. The bioactive aspects of EPS show value in pharmaceutical development because they possess antimicrobial and anti‐inflammatory abilities and antioxidant activity for bioremediation applications. As natural, biodegradable alternatives to synthetic polymers, microbial EPSs present promising opportunities for sustainable agricultural practices, environmental management, and therapeutic applications. Researchers should intensify their EPSs investigation because it enables them to harness this valuable material for resolving worldwide issues concerning food supply stability and ecological sustainability alongside climate tolerance.

## Author Contributions

All authors contributed to the study conception and design. **Ann Maxton:** conceptualization. **Aishmita Gantait** and **Sam A. Masih:** material preparation and first draft writing. **Adriano Sofo**, **Rosangela Addesso** and **Sam A. Masih:** writing – review and editing. All authors commented on previous versions of the manuscript. All authors read and approved of the final manuscript.

## Funding

This work was carried out within the Agritech National Research Center and received funding from the European Union Next‐GenerationEU (PIANO NAZIONALE DI RIPRESA E RESILIENZA (PNRR) ‐ MISSIONE 4 COMPONENTE 2, INVESTIMENTO 1.4 ‐ D.D. 1032 17/06/2022, CN00000022). This manuscript reflects only the authors' views and opinions, neither the European Union nor the European Commission can be considered responsible for them.

## Conflicts of Interest

The authors declare no conflicts of interest.

## Data Availability

Data sharing not applicable to this article as no datasets were generated or analysed during the current study.
